# Parent–Child Interaction Therapy for Children with Disruptive Behaviors and Autism: A Randomized Clinical Trial

**DOI:** 10.1007/s10803-022-05428-y

**Published:** 2022-01-25

**Authors:** Korrie Allen, John Harrington, Lauren B. Quetsch, Joshua Masse, Cathy Cooke, James F. Paulson

**Affiliations:** 1 Present Address: University of Denver, Denver, CO USA; 2 Children’s Hospital of the King’s Daughters, Norfolk, USA; 3 Eastern Virginia Medical School, Norfolk, USA; 4 Department of Psychological Science, University of Arkansas, 480 Campus Drive, Memorial Hall, Fayetteville, AR 72701 USA; 5 University of Massachusetts Dartmouth, Dartmouth, USA; 6 The Boston Child Study Center, Boston, USA; 7 Present Address: Old Dominion University, Norfolk, USA

**Keywords:** Parent–child interaction therapy, Autism spectrum disorder, Randomized clinical trial

## Abstract

A relatively large number of children with autism spectrum disorder (ASD) exhibit disruptive behavioral problems. While accumulating data have shown behavioral parent training programs to be efficacious in reducing disruptive behaviors for this population, there is a dearth of literature examining the impact of such programs across the range of ASD severity. To evaluate the effectiveness of Parent–Child Interaction Therapy (PCIT), an evidence-based treatment for children with problem behaviors and their families, in reducing disruptive behaviors among children (4–10 years) with ASD (without intellectual disabilities). Fifty-five children (85.5% male, 7.15 years; *SD* 1.72) were enrolled from pediatric offices and educational settings into a randomized clinical trial (PCIT: *N* = 30; Control: *N* = 25). PCIT families demonstrated a significant reduction in child disruptive behaviors, increase in positive parent–child communication, improvement in child compliance, and reduction in parental stress compared to the control group. Exploratory analyses revealed no differential treatment response based on ASD severity, receptive language, and age. Results are promising for the use of PCIT with children demonstrating disruptive behaviors across the autism spectrum.

## Introduction

Autism spectrum disorder (ASD), a continuum of neurodevelopmental disorders, is characterized by social deficits, communication impairments, and rigid, repetitive behaviors (APA, 2013). Over the past four decades, the estimated incidence of ASD in the United States has continued to grow with the latest prevalence rates standing at 1 in every 44 children (1 in 27 males, 1 in 116 females; Maenner et al., 2021). Since 2000, prevalence rates have increased by over 150% leading ASD to become an urgent public health concern (Maenner et al., 2021). Further, many of these children engage in aggressive and other disruptive behaviors (e.g., tantrums, self-injury) toward themselves, family members, peers, and teachers (Kaat & Lecavalier, 2013; Kanne & Mazurek, 2011) representing one of the most common reasons for referrals to mental health clinics and emergency departments (Pikard et al., 2018).

The presence of clinically significant behavioral problems among children with ASD is widely acknowledged and cited; however, the exact prevalence varies greatly due to the frequent use of clinical samples in studies (for a review, see Fitzpatrick et al., 2016; Hill et al., 2014; Solomon et al., 2008) and inconsistent or ambiguous definitions of behavior problems (see Hill et al., 2014; Tremblay et al., 2005). Some literature has reported that 50–70% of clinically-referred children and adolescents with ASD exhibited aggressive behavior to a caregiver, whereas other studies concluded that approximately 90% of study participants with ASD showed some form of challenging behavior (Jang et al., 2011; Kanne & Mazurek, 2011; Mazurek et al., 2013; McTiernan et al., 2011). One literature review concluded that approximately 25% of youth with ASD rise to the level of meeting diagnostic criteria for a disruptive behavior disorder (i.e., oppositional defiant disorder, conduct disorder; Kaat & Lecavalier, 2013). Comparatively, community samples of neurotypical children have indicated rates of persistently aggressive behavior ranging from 0.5 to 10% (Broidy et al., 2003; Lee et al., 2007). Even with the limitations in the literature, there is a clear concern for the prevalence of aggressive behaviors in the ASD population.

Importantly, the presence of behavior problems can yield a myriad of other consequences impacting both the child and the family. Namely, disruptive and aggressive behaviors can present barriers to learning (Murray & Farrington, 2010), assignment to residential or restrictive school placements (Dryden-Edwards & Combrinck-Graham, 2010), and further social impairment (Luiselli, 2009). Children with aggressive behaviors are at an increased risk for physical harm/safety concerns, reduced quality of life, increased familial financial strain, limited access to supports and services, and both contribute to and are a consequence of parental stress (Hodgetts et al., 2013; Kanne & Mazurek, 2011; Krahé et al., 2015). Failure to address behavioral problems in children with ASD during early- to mid-childhood allows these behaviors to become established; and without intervention, problem behaviors are unlikely to ameliorate (Emerson et al., 2014; Horner et al., 2002). The presence of behavioral problems among children with ASD impedes developmental progress and the acquisition of key skills emphasized by early intensive behavioral interventions (Jang et al., 2011). When behavioral problems are addressed and decreased, children with ASD are more likely to comply with more intense and focused therapies to address other ASD-related concerns (Masse et al., 2007).

### Treatment for Children with ASD and Disruptive Behaviors

#### Medication

Approximately two thirds of youth and adults with autism take psychotropic medication (Houghton et al., 2017). Although medication options for this population are limited, antipsychotics approved by the Food and Drug Administration (i.e., risperidone, aripiprazole) are frequently prescribed by providers to reduce irritability commonly associated with autism (Houghton et al., 2017). Studies have found as many as 50% of children with ASD are on at least one psychotropic medication (with rates often increasing with age) to treat non-core ASD symptoms including oppositional behaviors or aggression (Jobski et al., 2017; Ziskind et al., 2020). Although some studies have shown promising results when using these psychotropic drugs, adverse side effects are common and significant (e.g., exhaustion, rapid weight gain, anxiety, increased aggression; Larry & Erickson, 2018).

#### Psychosocial Treatments

As an alternative to medication, the intervention literature has strong support for the effectiveness of comprehensive services for children with ASD. Established behavioral and educational treatments are available, including Learning Experiences and Alternative Program for Preschoolers and their Parents, Treatment and Education of Autistic and Communication Handicapped Children (TEACCH Method), Early Start Denver Model, DIR/Floortime, Applied Behavioral Analysis (ABA), and ABA-derived models such as Early Intensive Behavioral Intervention (the UCLA Young Autism Project), Pivotal Response Treatment, and Positive Behavior Support (PBS; Carroll & Kodak, 2019; Masse et al., 2007; Smith & Iadarola, 2015). These focused therapies employ a number of techniques to increase socially-appropriate behaviors; decrease challenging behaviors; and improve language, social, and behavioral deficits in children with ASD (Carroll & Kodak, 2019). However, these therapies do not always involve direct parent coaching and require cooperative behavior from the child, which is problematic for children exhibiting oppositional behavior (Masse et al., 2007). Therefore, a behavioral intervention focused on reducing disruptive behaviors may act as a gateway treatment to more intensive interventions, or alternatively, may fulfill particular needs of families unable to access or afford more intensive, ABA-based treatments (McNeil et al., 2019; Williford et al., 2019). Due to many similarities between the behavioral problems exhibited by children with ASD and those displayed by neurotypical peers with challenging behaviors, it is appropriate to identify family-based evidence-supported treatments that could be translated to an ASD population to reduce disruptive behaviors and aggression, increase compliance, and improve overall family functioning (McNeil et al., 2019; Williford et al., 2019).

##### Parent–Child Interaction Therapy (PCIT)

Parent–Child Interaction Therapy is a two-phase, empirically-supported treatment designed for children ages 2–7 with disruptive behaviors (McNeil & Hembree-Kigin, 2010). Based on the attachment theory and social learning theory, PCIT places emphasis on improving the quality of the parent–child relationship and parent–child interactions. During the first phase, Child-Directed Interaction (CDI), parents are taught specific skills to enhance the parent–child relationship and increase positive parenting. During the second phase, Parent-Directed Interaction (PDI), parents are taught how to give effective commands and use consistent discipline techniques.

PCIT has demonstrated clinically significant improvements for families of children with disruptive behaviors and ASD. Specifically, positive outcomes have demonstrated enhanced interaction style of parents, decreased child behavior problems, improved child adaptability, increased child vocalizations, and higher child compliance (for a review, see Owen et al., 2019; Scudder et al., 2019). Moreover, similar outcomes on externalizing behavior, parenting skills, and parental stress have been found when matched-cases for children with and without ASD were compared (Parladé et al., 2019). Previous studies of PCIT with ASD have rarely explored children with more severe levels of autism, children’s medication use, or children outside the typical PCIT age range (older than 7 years; e.g., Scudder et al., 2019; see Owen et al., 2019 for a review).

PCIT is a unique treatment model for children with ASD and problem behaviors. Importantly, many of the therapies available to families of children with ASD and behavior problems are therapist-led intensive interventions whereas PCIT is a cost-effective time limited intervention designed to help parents address behavior problems. PCIT could possibly serve as a gateway therapy for more intensive treatments and be used in conjunction as a first-line treatment to prepare children with ASD for other comprehensive therapies (McNeil et al., 2019).

#### Additional Factors Impacting Treatment Effectiveness

##### ASD Severity

Although a strong research base is being built for conducting PCIT with children on the autism spectrum (see McNeil et al., 2019), most of the more methodologically rigorous studies for PCIT have been implemented with children with Level 1 severity (formerly Asperger syndrome; APA, 2013) or have only implemented components of the treatment (i.e., only CDI; see Owen et al., 2019). While some case studies have demonstrated PCIT’s success for children with more significant delays, there is a paucity of literature for determining the effectiveness of PCIT for children with lower levels of ASD functioning. More research is needed to determine the effectiveness of the entire PCIT protocol (i.e., both CDI and PDI phases) across the autism spectrum (McNeil & Quetsch, 2019).

##### Medication Use

Few PCIT studies with children with ASD have reported on or measured child medication use (Scudder et al., 2019). Yet, antipsychotic medications are prescribed at high rates to children with ASD (Ziskind et al., 2020). While the literature is limited, previous explorations of intensive behavioral interventions have shown improvements regardless of medication status; although, children taking antipsychotic medications may require fewer sessions to achieve behavioral goals (Frazier et al., 2010). A study controlling for medication use could further clarify the effectiveness of medication and/or PCIT on child behavioral outcomes.

##### Language

Individuals with ASD can experience communication deficits in both receptive and expressive language (Özyurt & Eliküçük, 2018). Due to the high demand for verbal comprehension inherent in PCIT, studies with PCIT have frequently limited the enrollment to children who have receptive language skills of at least 24 months (Beverly & Zlomke, 2019; Owen et al., 2019). Improvements in language for children with ASD have been demonstrated in previous PCIT studies (see Beverly & Zlomke, 2019). However, few studies in PCIT have explored how language may impact treatment outcomes, thus warranting further investigation.

##### Age

Children with ASD may present with disruptive and aggressive behaviors but fall outside of the standard age range of PCIT. Given that more than half of children with ASD may demonstrate cognitive capabilities lower than their chronological age, expanding PCIT’s age range may help address the children who are developmentally delayed but who may have otherwise aged-out of early intervention services to target problem behaviors (Charman et al., 2011; Maenner et al., 2021). Only a few PCIT studies have expanded the age range up for exactly this reason (e.g., 8-year-olds: Zlomke et al., 2017; 12-year-olds: Solomon et al., 2008). Understanding the impact of PCIT on children with differing profiles and a broader age range will help inform future studies conducted with this population.

### Purpose and Hypotheses

The purpose of this study was to evaluate the effectiveness of PCIT in reducing oppositional behaviors and increasing positive parenting behavior among children (4 to 10 years) with ASD (without intellectual disabilities). This study expands the PCIT effectiveness research by including children with varying levels of ASD and who may fall outside the standard age-range for PCIT. The study included three hypotheses: (1) PCIT will result in a significant decrease in parent-reported disruptive behaviors; (2) parent and child interactions (child compliance rates and parenting skills) will significantly improve over the course of PCIT; and, (3) PCIT will result in significant improvements in parent efficacy and parent mental health (stress and depression). The second goal of the study was to perform exploratory analyses to assess the differential impact of the full PCIT protocol by autism severity, medication use, and language level on the disruptive behaviors of children across the autism spectrum. Finally, this study assessed parental satisfaction with PCIT.

### Research Design

The study design followed a step-wise model for conducting psychosocial interventions for ASD, as outlined by Smith et al. (2007) and in accordance with the guidelines adopted by the National Institute of Mental Health (NIMH). This research design adheres to the recommendation that when applying PCIT to a new population, it should first be empirically tested in its standard form to determine its efficacy before any modifications are made to the model (Masse et al., 2007; McCabe et al., 2005). Thus, our study evaluated the efficacy of PCIT in its manualized form (Eyberg & Funderburk, 2011) for children with ASD and behavioral problems.

## Method

### Participants

Families were recruited from the eastern United States. The region included 10 cities and 6 counties in two states located in socio-economically and culturally diverse areas that ranged from rural to urban and suburban settings. The region consisted of 61% White, 31% Black, and 8% other ethnic groups. Fifty-five female and eight male caregivers (*N* = 55 families) and their 4- to 10-year-old children participated in the present study. All adult caregivers living in the home were encouraged to participate in this treatment as research suggests that dual-parent involvement (e.g., mother, father) leads to better maintenance of treatment gains (Bagner & Eyberg, 2003). Although eight fathers participated in treatment, the primary caregivers in each household were identified as the participating mothers. Therefore, data for this study only include information from the primary caregivers (i.e., mothers, in this case).

Children who participated in the study (Table 1) were mostly boys (85.5%) with a mean age of 7.15 years (*SD* 1.72). Their racial/ethnic composition was 65.5% White, 16.4% Black, 9.1% Latinx, 9.1% other ethnicity. Most children (80%) were referred by pediatric health care professionals, 12% were referred by teachers, and 8% were self-referred. At the time of intake, 56.4% were prescribed medication to address behavioral issues. Most children came from families with total household incomes between $50,000 and $99,000 (*n* = 29; 52.7%). To be included in the study, children had to demonstrate at-risk or clinically significant externalizing behaviors (Behavior Assessment Scale for Children [BASC]; Reynolds & Kamphaus, 1992), be diagnosed with ASD by a health professional prior to the study (confirmed and assessed for severity using the Child Autism Rating Scale [CARS]; Schopler et al., 1980), and obtain a receptive language age equivalent of 2 years or higher (Peabody Picture Vocabulary Test [PPVT]; Dunn & Dunn, 2007) to ensure the child was able to follow basic parental commands. Parents were asked to report if the child had been previously diagnosed with an intellectual disability (*yes/no*). No parents endorsed concerns which was confirmed through a check of the children’s medical records. However, no cognitive data was collected in the present study.

**Table 1:** Demographic composition of sample

	Treatment group^a^ *M(SD)* or *N*(%)	Control group^b^ *M(SD)* or *N*(%)
Child sex		
Male	26 (86.7%)	21 (84.0%)
Female	4 (13.3%)	4 (16.0%)
Child age	7.03 (1.6)	7.26 (1.4)
Child ethnicity		
White	17 (56.7%)	19 (76.0%)
Black	7 (23.3%)	2 (8.0%)
Latinx	4 (13.3%)	1 (4.0%)
Other	2 (6.7%)	3 (12.0%)
Family financial status		
Less than $25,000	2 (6.7%)	3 (12.0%)
$25,000–$49,999	7 (23.3%)	5 (20.0%)
$50,000–$99,000	16 (53.3%)	13 (52.0%)
Over $100,000	5 (16.7%)	4 (16.0%)
BASC T-score		
Externalizing	74.8 (11.1)	73.2 (11.5)
CARS-2 T-score	49.9 (9.5)	48.3 (8.9)
PPVT standard score	90.3 (8.7)	94.7 (7.2)
Psych Rx	16 (53.3%)	15 (60.0%)

*N*^a^ = 30, *N*^b^ = 25

*BASC* behavior assessment system for children, *CARS* Childhood Autism Rating Scale, *PPVT* peabody picture vocabulary test, *Psych Rx* psychological prescription/medication

**Fig. 1:**
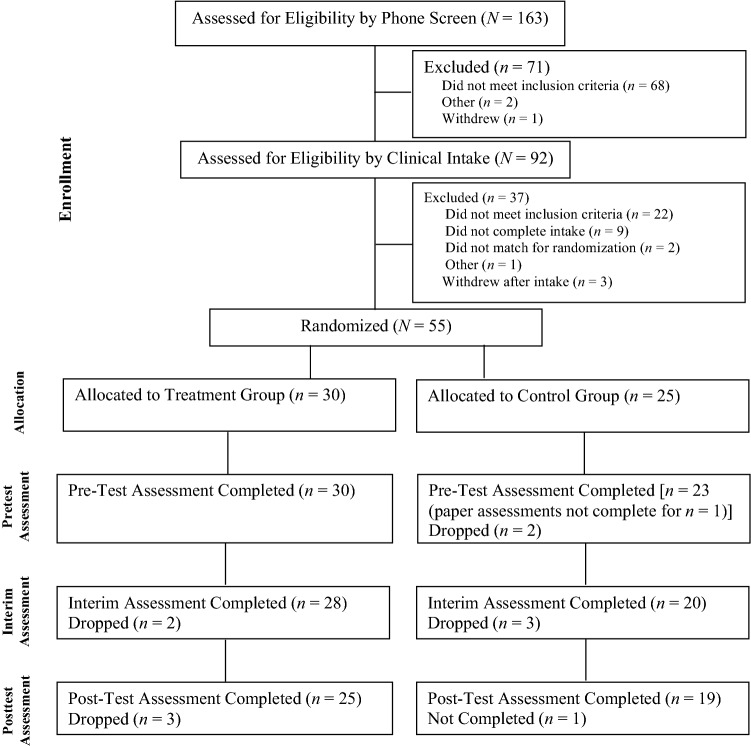
CONSORT diagram

Overall, 181 interested families responded to study recruitment. Please see Fig. 1 for an overview of the screening and recruitment process. Recruitment took place over 10 months, and assessment and treatment completion took 18 months. In total, 163 phone screens were conducted and 92 families qualified and were scheduled for a clinical intake assessment. Of those families that completed the intake process, 55 met criteria for inclusion. Families were excluded if based on the screening measures the child (a) had limited receptive language (*n* = 8), (b) lacked severe behavior problems (*n* = 10), (c) were not previously diagnosed with ASD by a health professional (*n* = 4), or (d) the family did not complete the intake process (*n* = 9). There were three families screened as eligible for the study that cancelled their participation from the original group. Families not meeting the inclusion criteria (*n* = 37) were given feedback and appropriate recommendations for services. Upon completing the clinical intake, children were stratified based on the dichotomized variable of psychiatric medication use (yes/no), then matched using the continuous variables of externalizing behaviors (BASC), severity of autism (as measured using the CARS), and age. Families were assigned using stratified randomization to the control or treatment (PCIT) group using a research randomizer program. Randomization was determined by the lead researchers (first and second authors). Two children did not match and were not included in the randomization process, yielding a total of 30 treatment group (TG) families and 25 control group (CG) families. Treatment for children with ASD in the study location was limited. Many families were on a wait-list of 12–18 months for ABA. Families in both the TG and CG continued routine community care throughout the study, which primarily consisted of speech therapy services (TG = 38%, CG = 40%).

### Procedure

The study was approved by the Eastern Virginia Medical School and the Children's Hospital of the King's Daughters Institutional Review Boards. Children were recruited by providing study information packets to pediatric offices, the Tidewater Autism Society of America, Community Service Boards, and school systems. Children were screened prior to enrollment on level of disruptive behavior (BASC), autism severity (CARS), and receptive language (PPVT). Enrolled families completed a baseline assessment (Time 1: pre-treatment) that included parental report questionnaires and parent–child observations which were videotaped and coded by research personnel. Approximately 8 weeks after the Time 1 assessment, all TG and CG families were scheduled to complete an interim-treatment assessment (Time 2). This interim assessment marked the transition from CDI to PDI for treatment families. The Time 3 assessment occurred post-treatment (upon graduation for TG), 8 weeks after Time 2. All families were compensated for study participation at Time 1 and Time 3 assessment visits. Data collection and treatment delivery was conducted in a medical center. Enrollment, assessments, and treatment delivery were conducted by the research team.

### Screening Measures and Inclusion Measure

#### Behavior Assessment Scale for Children-Second Edition (BASC-2)

The BASC-2 (Reynolds & Kamphaus, 1992) was used in the identification and differential diagnosis of emotional/behavioral disorders in children. Child participants had to receive an at-risk score (T ≥ 60) or higher on externalizing behavior problems to meet eligibility criteria for the present study. The Externalizing subscale of the measure was also used to determine if any changes in child behavior were detected over time.

#### Childhood Autism Rating Scale, Second Edition (CARS-2)

The CARS-2 (Schopler et al., 1980) was used on the child sample to identify and confirm a diagnosis of autism while distinguishing them from children with other developmental disabilities. The measure is empirically validated and provides concise, objective, and quantifiable ratings based on direct behavioral observation. Only children with an existing diagnosis of ASD were included in the present study. Outcomes of the CARS-2 produce a cutoff score (indicative of autism > 28) with scores above this being further identified as either “mild to moderate” (*T* scores between 29 and 49) or “severe” (scores at or above a *T*-score of 50). None of the children in the sample had intellectual disabilities; therefore, the CARS-2 Standard Form was delivered for children 6 years of age and younger unless the child 7 or older had a notable communication impairment. The CARS-2 High Functioning was delivered to youth 7 years of age or older in the sample. Children’s CARS-2 *T* scores ranged from 32 to 65, with the mean score of 48.3 (*SD* 8.3).

#### Peabody Picture Vocabulary Test, Fourth Edition (PPVT-IV)

The PPVT-IV (Dunn & Dunn, 2007) is an individually administered, untimed, norm-referenced, wide-range test designed for children and adults ages 2.6 to 90 + years that assesses receptive vocabulary and verbal ability. The PPVT-IV has measures of reliability in the 0.90’s and validity studies indicate it is sensitive enough to identify language-delayed students. Children met participation criteria if their receptive language score was at or above a 2-year-old equivalent.

### Outcome Measures of Child and Parent Functioning

Primary outcome measures included measures of externalizing behavior and observational measures. Secondary outcome measures included assessments of parenting stress, depression, and locus of control.

#### Eyberg Child Behavior Inventory (ECBI)

The ECBI (Eyberg & Boggs, 1989) is a 36-item parent-report scale of disruptive behavior and includes two scales: Intensity and Problem. The Intensity Scale measures the frequency with which disruptive behavior occurs using a 7-point Likert-type scale (1 = *never* to 7 = *always*). The Problem Scale includes “yes” or “no” responses and measures how problematic the child’s behavior is for the parent. The Intensity and Problem scales yield test–retest reliability coefficients of .80 and .85 across 12 weeks, respectively, and .75 across 10 months. The ECBI has been normed for children with ASD (ages 2–12 years) with cutoff scores on the Intensity (x = 169) and Problem Scales (x = 23) being significantly higher than neurotypical comparisons (i.e., x = 132, 15, respectively; Jeter et al., 2017). The ECBI was administered to both groups at each assessment period (Times 1–3) and weekly to the TG families.

#### Dyadic Parent–Child Interaction Coding System (DPICS)

The DPICS (Eyberg et al., 2004) is a behavioral observation coding system that measures parental verbalizations (i.e., labeled and unlabeled praise, behavior descriptions, reflections, imitation, neutral talk, questions, direct and indirect commands, criticism) and child compliance. It acts as a measure of the quality of parent–child interaction during three 5-min standard situations (i.e., Child-led Play, Parent-led Play, Clean-up) that vary in the degree of parental control. While Child-led Play (CLP) assesses parents’ use of skills that allow the child to lead an interaction, Parent-led Play (PLP) instructs the parent to lead and have the child follow, while Clean-up (CU) requires the child to put away all the toys without assistance from the parent. Compliance in this study represents the average compliance rate of PLP and CU at each assessment point. The DPICS was administered weekly to the TG families. Frequency counts of each of the “Do” (i.e., labeled praises, behavior descriptions, reflections) and “Don’t” (i.e., questions, direct and indirect commands, criticism) skills were gathered in a 5-min observation period at the outset of each session. Competency was reached when a parent attained 10 labeled praises, 10 behavioral descriptions, 10 reflections, with 3 or less “Don’t” skills combined during the 5-min coding period.

Researchers received extensive (40 h) DPICS training to ensure reliability. Coders were considered reliable after attaining a .75 kappa for each of the “Do” and “Don’t” skills on five consecutive observations. Throughout the study, two researchers blinded to group assignment independently observed the same individuals for 50% of all sessions and maintained a .85 inter-rater reliability.

#### Parent Stress Index-Short Form (PSI-SF)

The PSI-SF **(**Abidin, 1995) is a 36-item parent self-report measure of stress as it relates to in the parent–child dyad with strong reliability and validity indices. The Total Stress score was the only scale utilized in the present study.

#### Parenting Locus of Control-Short Form (PLOC-SF)

The PLOC-SF (Campis et al., 1986) is a 25-item self-report questionnaire that measures the degree to which parents feel in control of their child’s behavior.

#### Beck Depression Inventory (BDI-II)

The BDI-II (Beck et al., 1961) is a 21-item self-report measure assessing the intensity of depression. Respondents are asked to consider how they have been feeling over the last two weeks and respond to specific items about depression-related symptoms on a scale from 0 to 3. Higher scores on the BDI-II indicate greater severity of depression.

### Measure of Treatment Satisfaction

#### Therapy Attitude Inventory (TAI)

The TAI (Eyberg, 1993) is a 10-question measure containing items on a 5-point Likert scale. Higher scores represent higher levels of caregiver satisfaction. The measure addresses the impact of parent training skills on such areas as confidence in discipline skills, quality of parent–child interaction, the child’s behavior, and overall family adjustment. The TAI was administered at Time 2 and Time 3 assessments to TG families only.

### Treatment

PCIT sessions were conducted by a clinical psychologist once a week and lasted between 60 and 90 min. Families in the TG condition received the entire protocol of PCIT (i.e., both CDI and PDI phases) unless families terminated before treatment completion. In both phases of treatment (CDI, PDI), therapists actively coached parents toward understanding of the therapeutic interaction skills as assessed during a 5-min parent–child observation (DPICS) at the start of the session. On average, families achieved CDI skills competencies in 6.2 sessions and PDI in 5.9 sessions. Throughout treatment, parents were asked to practice the skills at home daily in 5–10 min sessions, initially focusing on CDI skills and then incorporating PDI skills at times when a command was necessary. The therapists included a licensed clinical psychologist and a supervised post-doctoral clinical psychology fellow, each of whom attended a 40-h PCIT training conducted by a PCIT Global Trainer. All therapy sessions were videotaped, and 50% of the session tapes from each family were randomly selected and checked independently by two coders for integrity using the PCIT treatment manual checklist. Accuracy was 95% with the treatment protocol. In addition, supervision from a PCIT Global Trainer was received regularly.

### Data Analysis

Mean scores on parent-report questionnaires as well as behavioral observation counts and ratios derived from the DPICS were primarily analyzed using Repeated Measures MANOVAs. This is a suitable technique given the uniformity of the assessment schedule. Additionally, it is preferable to the alternative of multiple univariate tests as it detects patterns between multiple dependent variables which may not otherwise arise in univariate tests. Additional univariate comparisons are presented as follow-ups to the MANOVAs, as are graphical displays of observed effects.

## Results

Among the 30 TG families and 25 CG families that were initially enrolled in study, 25 TG and 19 CG families completed all three assessment time points (Time 1–3) in the study (see Fig. 1). Forty-four families completed the study yielding an attrition rate of 19 percent. The primary reason for dropout was relocation to a new city. Overall, the dropout rate is significantly lower than would be expected considering that attrition rates from child psychotherapy range from 40 to 60% (Wierzbicki & Pekarik, 1993). Significant differences were found between groups on all primary outcome measures from Time 1 to Time 3 (see Table 2).

**Table 2:** Outcome measures

Measure	Group	Pretreatment	Interim	Posttreatment	Pre-to-post Δ	|d*|	*df*	*F(p)*
	*N (44)*	*M (SD)*	*M (SD)*	*M (SD)*	*M (SD)*			
Primary measures								
ECBI—intensity	CG	153.6 (22.7)	142.6 (27.94)	139.0 (25.5)	− 16.2 (18.8)	2.04	40	38.18 (< .001)
	TG	158.7 (25.8)	114.5 (33.3)	91.1 (27.8)	− 68.5 (32.1)			
ECBI—problems	CG	19.6 (4.96)	17.4 (7.79)	14.6 (8.20)	− 4.27 (4.62)	1.09	40	10.51 (.002)
	TG	20.2 (7.18)	12.2 (8.25)	7.81 (8.60)	− 12.91 (10.27)			
BASC- externalizing problems	CG	73.2 (11.5)	65.2 (11.0)	65.6 (11.7)	− 5.42 (6.28)	1.52	39	22.88 (< .001)
	TG	74.8 (11.1)	63.9 (10.2)	60.2 (8.14)	− 16.86 (8.63)			
DPICS “Do” Behaviors	CG	3.59 (3.02)	4.56 (3.48)	4.68 (3.47)	1.35 (3.59)	3.30	40	94.31 (< .001)
	TG	5.20 (3.95)	26.88 (12.29)	35.52 (12.35)	30.32 (11.89)			
DPICS “Don’t” Behaviors	CG	19.47 (15.55)	21.22 (9.25)	21.53 (12.56)	2.82 (9.46)	2.17	40	47.20 (< .001)
	TG	19.8 (9.29)	4.25 (7.81)	1.56 (2.52)	− 18.24 (9.94)			
DPICS PDP compliance	CG	.474 (.270)	.384 (.286)	.471 (.230)	− .021 (.169)	1.87	39	32.25 (< .001)
	TG	.335 (.195)	.332 (.238)	.727 (.209)	.393 (.264)			
DPICS cleanup compliance	CG	.555 (.245)	.594 (.249)	.505 (.135)	− .054 (.224)	.98	40	8.85 (.005)
	TG	.465 (.285)	.390 (.236)	.731 (.315)	.266 (.403)			
Secondary measures								
Parenting stress total	CG	114.4 (21.6)	114.5 (19.1)	109.7 (22.1)	− 8.81 (15.59)	.82	39	5.95 (.019)
	TG	122.7 (23.1)	109.3 (23.2)	96.6 (28.2)	− 27.7 (28.22)			
Beck depression inventory	CG	11.0 (5.39)	9.11 (8.00)	7.00 (6.63)	− 4.33 (3.91)	.42	41	1.64 (.208)
	TG	15.3 (7.57)	11.8 (9.46)	8.16 (8.19)	− 7.16 (8.74)			
Parenting locus of control	CG	49.5 (10.3)	48.1 (10.4)	47.2 (11.6)	− 4.00 (8.70)	.57	41	3.40 (.072)
	TG	52.3 (7.78)	48.0 (10.0)	44.2 (8.98)	− 8.92 (8.59)			

*CG* control group, *TG* treatment group, *ECBI* eyberg child behavior inventory, *BASC* behavior assessment system for children, *DPICS* dyadic parent–child interaction coding system, *DPICS “Do” Behaviors* behavior descriptions, reflections, labeled praises. *DPICS “Don’t” Behaviors* questions, commands, criticism

^*^Effect sizes of *d* > .80 are considered large

### Change in Child Disruptive Behavior

**Fig. 2:**
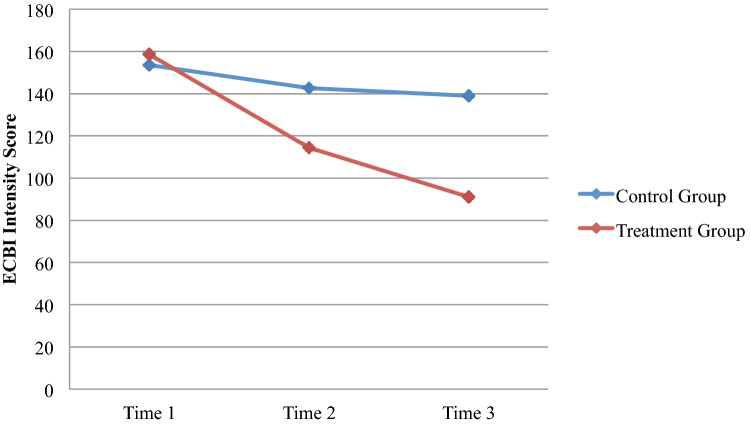
Change in ECBI intensity

The two parent-report measures that were used to assess disruptive and oppositional behavior observed by the parent in the home included the ECBI (Table 2; Fig. 2) and the BASC (Table 2). On the ECBI Intensity scale, a significant Time X Group interaction was observed, such that children in the TG demonstrated a much steeper decline in behavioral intensity (Wilk’s *λ* (2, 39) = 47.28,* p* < .001; Partial *η*^*2*^ = .48). Univariate tests reveal that children receiving PCIT demonstrated lower intensity of behavior problems at Time 2 (*F*(1,39) = 8.21,* p* = .006) and significantly increased that difference (*F*(1,39) = 30.76,* p* < .001) at Time 3 (see Fig. 2) compared to CG families. A similar interaction was observed for the ECBI Problems scale (Wilk’s *λ* (2, 37) = 5.77,* p* = .007; Partial *η*^*2*^ = .238), revealing that children in the TG demonstrated significantly fewer problems at Time 3 (*F*(1,37) = 8.41,* p* = .006), but not at Time 2. Overall, each child in the treatment group scored in the non-clinical level of the ECBI on both the Problems and Intensity scale at the conclusion of the treatment.

When measured with the BASC Externalizing Problems subscale (Table 2), a similar Time X Group interaction was observed, such that children in the TG demonstrated continued decline in externalizing problems at Times 2 and 3, whereas the CG leveled off after Time 2 (Wilk’s *λ* (2, 36) = 11.79,* p* < .001; Partial *η*^*2*^ = .40). Univariate tests revealed that children in the TG demonstrated lower levels of Externalizing Problems at Time 3 (*F*(1,37) = 6.61, *p* = .014) but not at Time 2.

### Change in Parent–Child Interactions

**Fig. 3:**
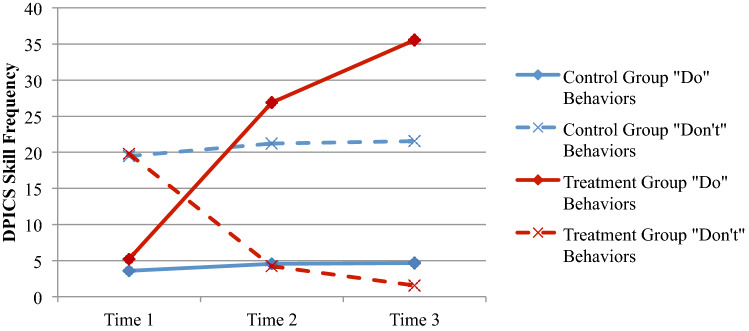
Average DPICS skill frequency

In terms of PCIT “Do” skills (measured by DPICS labeled praises, behavior descriptions, reflections), parents in the TG showed significant increases over the three assessment periods (Wilk’s *λ* (2,37) = 49.81,* p* < .001; Partial *η*^*2*^ = .73). Univariate tests indicated no statistically significant differences between groups at baseline with TG parents showing significantly more “Do” behaviors at Time 2 (*F*(1,38) = 49.07,* p* < .001) and maintaining this change (*F*(1,48) = 299.19,* p* < .001) through Time 3 (See Fig. 3).

PCIT “Don’t” skills (DPICS commands, questions, criticism) showed a similar but inverse pattern over time. Parents in the TG showed significant decreases in “Don’t” behaviors over the three assessment periods (Wilk’s *λ* (2, 37) = 21.33,* p* = .001; Partial *η*^*2*^ = .54). Univariate tests indicated no statistically significant differences between groups at Time 1, with TG parents showing significantly fewer “Don’t” behaviors at Time 2 (*F*(1,38) = 36.68,* p* < .001), and maintaining this change (*F*(1,38) = 63.53,* p* < .001) through Time 3 (Fig. 3).

Parents and children were observed for compliance/command ratio during the PLP and CU periods of the DPICS (Table 2). Treatment families showed a statistically significant Time X Compliance ratio change during the PLP activity (Wilk’s *λ* (2, 36) = 15.85,* p* < .001; Partial η^2^ = .47) with significant relative improvement occurring at Time 3 (*F*(1,37) = 21.22,* p* < .001). A very similar pattern was observed during the CU activity with the Time X Compliance being significant (Wilk’s *λ* (2, 37) = 7.99,* p* = .001; Partial *η*^*2*^ = .30) and the major improvement being observed for Time 3 (*F(*1, 38) = 10.07,* p* = .003).

### Change in Parenting Stress and Psychopathology

**Fig. 4:**
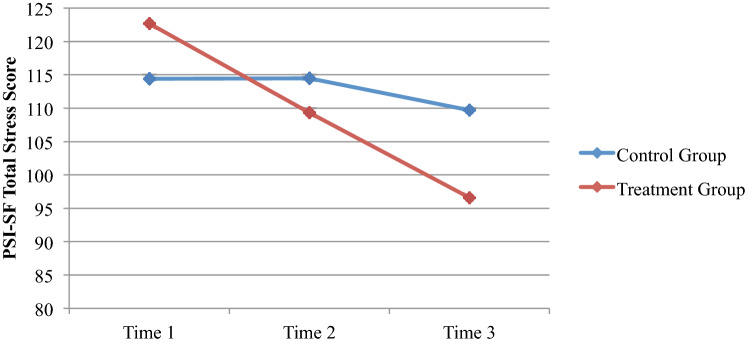
Change in parent stress total score

The parent-level variables revealed a large variation, with only parental stress demonstrating a statistically significant change between the TG and CG. On the PSI-SF Total Stress scale, a significant Time X Group interaction was observed, such that parents in the TG demonstrated a steeper decline in parenting stress over the course of the study (Fig. 4; Wilk’s *λ* (2, 37) = 4.67,* p* = .016; Partial *η*^*2*^ = .20). No significant difference were found between the TG and the CG on the PLOC-SF (*p* = .072) or BDI-II (*p* = .208; Table 2).

### Exploratory Analyses

#### Autism Severity

All TG children were divided into ASD severity groups according to CARS-2 scores at or above a *T*-score of 50. In terms of ECBI Intensity scores, the most sensitive indicator of behavior change in this study, there was not an ASD Severity X Time effect. This appears to indicate children with varying severity of ASD responded similarly to the treatment over time (NS, Partial *η*^*2*^ = .25, *p* = .16).

#### Medication Use

On the ECBI Intensity scale, no significant differences were found between children on psychiatric medications and those not on psychiatric medications, regardless of whether they were in the TG or CG. Medication X Group X Time was not significant (NS, Partial *η*^*2*^ = .08, *p* = .076).

#### Receptive Language and Age

Pearson’s r correlations were conducted for the TG to determine if children’s receptive language (PPVT-IV) or age impacted change in child behavior (post-treatment ECBI Intensity—pre-treatment ECBI Intensity). The findings were not significant.

### Treatment Satisfaction

Findings on the TAI show that TG parents found the techniques helpful in regards to disciplining their child (91%) and teaching their child new skills (93%). All parents of children in the TG felt their relationship with their child was better than before the program and that their child’s behavior problems and compliance with parental commands and requests had improved. Ninety-three percent of the parents reported satisfaction with the progress their child had made in regards to their general behavior and many (86%) felt that the program helped with other general personal and family problems. These findings suggest that families of children with a range of ASD presentations were highly satisfied with PCIT.

## Discussion

This study further demonstrates the efficacy of PCIT among children with ASD and co-occurring behavior problems. The treatment was provided to children with ASD (without intellectual disabilities) and the results demonstrated similar behavioral changes to children without ASD who received PCIT. Specifically, based on both parent-report and observation data, children in the TG demonstrated a significant reduction in challenging behavior at the completion of CDI, and this reduction continued through the completion of PDI. Furthermore, from Time 1 to Time 3, TG families used significantly more relationship-building skills and obtained more compliance from their children when giving them commands, compared to the CG. This finding is similar to other PCIT literature conducted with children with ASD (Ginn et al., 2015; Masse et al., 2016; Parladé et al., 2019; Scudder et al., 2019; Zlomke et al., 2017).

The overall quality of the parent–child relationship significantly improved as well. Parents completing PCIT demonstrated a significant improvement in differential attention to their children’s behaviors through describing their children’s actions, reflecting their words, and giving them labeled praises for appropriate behaviors. Parents in the TG also demonstrated a significant reduction in the use of commands, questions, and criticisms when interacting with their children. Importantly, parents progressed in issuing effective commands to their children and following through appropriately, resulting in improvements in child compliance and decreases in parent–child conflict.

Secondary analyses were conducted examining parental stress and psychopathology. The TG demonstrated significant change in parenting stress over the course of the study, but treatment did not change parental perceptions of control or ratings of depression. It is well-established that there is significant parental stress associated with parenting a developmentally delayed child (e.g., Lichtlé et al., 2020; Padden & James, 2017); yet, prior PCIT research examining parental stress with the ASD population has been mixed (Agazzi et al., 2017; Solomon et al., 2008). As such, given the varying levels of ASD severity in this study, this finding is promising.

In addition, due to the limited availability of services for children with ASD in the study region, children between 4 to 10 years of age with varying levels of ASD severity were included in the study. The results indicate that despite the slightly higher age range (*M* = 7.18 years) and range of ASD severity, the TG responded to PCIT in similar ways to children without ASD (Boggs et al., 2004). Children were also stratified by psychiatric medication status before being randomly assigned to the TG or the CG. Medication use had no significant impact on child disruptive behaviors while PCIT did have a clear and significant impact on reducing disruptive behaviors for children in the TG. Additionally, associations of disruptive behavior change with receptive language functioning and with child age were explored for the TG. Outcome indicated that age and language differences were not associated with difference in behavior change signifying that PCIT resulted in similar changes regardless of age and receptive language.

Families also reported that PCIT was an effective and satisfactory treatment for their children’s behavior problems: 90% of the TG families reported satisfaction with the process and outcome of treatment, and over 85% felt it improved their parenting skills, their child’s behavior, and the overall family functioning. Although attrition in child therapy has been identified as a substantial problem (NIMH, 2001), few in this study dropped out of treatment.

PCIT was used in its original form with only tailoring, as suggested by the creator of PCIT, Sheila Eyberg, to meet the needs of individual families (2005). The theoretical and empirical foundation of PCIT was maintained, along with its core defining features. Outcomes from the present study are in line with previous research claims positing that PCIT can be effective without significant modifications for children with developmental delays, including children in the older range of typical PCIT research (ages 7–10 years; McDiarmid & Bagner, 2005).

### Implications for Research, Policy, and Practice

Previous studies evaluating the impact of autism treatment approaches have been conducted with middle- to upper-middle-class families with an estimated annual treatment cost ranging from $25,000 to $60,000 per child and requiring up to 25 h per week (Solomon et al., 2007). For many families, there is a lack of both treatment availability and financial resources (Mackintosh et al., 2012). This study demonstrates that PCIT offers an innovative, more cost-effective approach to delivering an evidence-based therapy to a diverse population. Specific benefits of this model of treatment include: (a) a family-based approach that addresses caregivers’ capacity to manage ASD-related behaviors; (b) direct-coaching to maximize parental learning and retention; (c) a time-limited model; and (d) a treatment model that can be wildly disseminated. Historically, few studies of behavioral treatment of ASD employed an experimental design (2 of 68 studies per a 2001 meta-analysis; Lord & McGee, 2001); however, even though recent studies for various early interventions are utilizing randomized controlled trials, only a few of these are behaviorally-based treatments. Additionally, many studies have substantial bias and limitations preventing robust findings for this population (French & Kennedy, 2018; Tachibana et al., 2017). Therefore, the use of a controlled randomized design for this study addresses a significant gap in the research for this treatment and population.

Treatment research for children with ASD has primarily focused on the benefits of early intensive behavioral intervention (e.g., Remington et al., 2007; Rogers & Vismara, 2008). Many children receive some form of intensive behavioral training after being diagnosed between the ages of 3 to 4; by age 5, the treatment options begin to significantly decrease. However, the majority of children with ASD continue to experience language, social, and behavioral difficulties throughout their school years (Marsh et al., 2017; McKean et al., 2017). Additionally, for families receiving later diagnoses or who are limited by accessibility of resources (Godon-Lipkin et al., 2016), PCIT may be a gateway to reduce disruptive behaviors, improve the effectiveness of other interventions, and increase accessibility due to wide availability of PCIT providers in the United States (Scudder et al., 2017; Soke et al., 2018). A research review (Solomon et al., 2008) concluded that children with autism are significantly at-risk for problematic behaviors which, without intervention, are more likely to worsen than improve. Despite this problem, our understanding of effective behavioral treatment of children with ASD is limited. PCIT may be one answer.

### Limitations

Future studies may strive to overcome the present study’s limitations. For example, the study did not include an alternative treatment control; therefore, the results must still be considered provisional. Additionally, future research on PCIT may benefit from further analyses regarding how treatment may differentially impact children at various levels of autism functioning. Researchers should focus on PCIT adaptations for children at lower levels of functioning. Moreover, while the present study was unique in that it included youth classified as severely autistic, it excluded youth with a comorbid intellectual disability. Future studies may also benefit from measuring, reporting, and controlling for youth’s cognitive functioning (e.g., IQ) to lend insight in PCIT’s effectiveness of youth with ASD and diverse intellectual levels.

Although this study did not measure non-disruptive behaviors of autism, such as self-stimulation, eye contact, language, and social engagement, positive changes in these behaviors were both observed by therapists and reported by parents and teachers. Next steps include assessing these behaviors in the context of other child characteristics including autism severity, language level, and cognitive functioning.

As PCIT did not significantly decrease parent depression, future studies should explore ways to improve these measures of parent-functioning, possibly through the addition of a parent psycho-education/treatment module or referral for individual parental therapy. A larger sample size would allow for more thorough analyses of other possible correlates, such as age and gender.

In families with typically-developing children, PCIT has been found to provide treatment effects lasting up to two years (Boggs et al., 2004). In order to determine whether the treatment gains demonstrated by families completing PCIT will be maintained, follow up data has been collected three months after treatment completion. The results of these data will be reported in future publications. Furthermore, in-depth analyses of PCIT’s impact on child language from this study will also be presented in future publications.

Based on the results of this study, PCIT should be considered a viable treatment for children with ASD and behavioral problems (without intellectual disabilities). In addition, the therapy can effectively prepare children for other intense and focused ASD therapies requiring cooperation and attention (Masse et al., 2007; Williford et al., 2019) and take advantage of needed therapies (e.g., speech, occupational therapy) when behavioral problems limit their ability to engage in the therapeutic process. Parents were satisfied with their experience with PCIT and reported significant improvement in child behavior, compliance, and parent–child interactions. This study also expands the limited research on PCIT among children with autism by improving the generalizability to children of varying autism severity.
